# Transcriptome Analysis Reveals Strain-Specific and Conserved Stemness Genes in *Schmidtea mediterranea*

**DOI:** 10.1371/journal.pone.0034447

**Published:** 2012-04-04

**Authors:** Alissa M. Resch, Dasaradhi Palakodeti, Yi-Chien Lu, Michael Horowitz, Brenton R. Graveley

**Affiliations:** 1 Department of Genetics and Developmental Biology, Stem Cell Institute, Health Center, University of Connecticut, Farmington, Connecticut, United States of America; 2 Institute for Stem Cell Biology and Regenerative Medicine, National Center for Biological Sciences, Bangalore, India; 3 Department of Pathology and Laboratory Medicine, Weill Cornell Medical College, New York, New York, United States of America; German Cancer Research Center (DKFZ) and Heidelberg University, Germany

## Abstract

The planarian *Schmidtea mediterranea* is a powerful model organism for studying stem cell biology due to its extraordinary regenerative ability mediated by neoblasts, a population of adult somatic stem cells. Elucidation of the *S. mediterranea* transcriptome and the dynamics of transcript expression will increase our understanding of the gene regulatory programs that regulate stem cell function and differentiation. Here, we have used RNA-Seq to characterize the *S. mediterranea* transcriptome in sexual and asexual animals and in purified neoblast and differentiated cell populations. Our analysis identified many uncharacterized genes, transcripts, and alternatively spliced isoforms that are differentially expressed in a strain or cell type-specific manner. Transcriptome profiling of purified neoblasts and differentiated cells identified neoblast-enriched transcripts, many of which likely play important roles in regeneration and stem cell function. Strikingly, many of the neoblast-enriched genes are orthologs of genes whose expression is enriched in human embryonic stem cells, suggesting that a core set of genes that regulate stem cell function are conserved across metazoan species.

## Introduction

The freshwater planarian *Schmidtea mediterranea* has emerged as an important model organism for studying stem cell biology and regeneration, because of its' ability to completely regenerate following injury [1], [2]. The regenerative capability of adult planarians is facilitated by a population of adult pluripotent stem cells called neoblasts, which have the ability to undergo self-renewal and differentiate into all cell types of the animal, including germ cells. *S. mediterranea* is a non-parasitic flatworm in the phylum Platyhelminthes, a clade of metazoans largely unexplored using genomic approaches. While a draft assembly of the *S. mediterranea* genome, which contains four chromosomes, has been generated [3] and EST sequencing projects have characterized thousands of *S. mediterranea* transcripts [4], [5], characterization of the planarian transcriptome remains incomplete. Recent analyses have more thoroughly interrogated the *S. mediterranea* transcriptome using RNA-Seq and identified a significantly larger fraction of transcribed sequences in the genome [6], [7], [8]. However, none of these studies examined the dynamics of gene expression in different strains or in different cell types.

Sexual and asexual strains of *S. mediterranea* exist in nature, and sexual animals can regenerate both ovaries and testes, making *S. mediterranea* an attractive system for studying germline development [1]. The sexual strain is a cross-fertilizing hermaphrodite, while the asexual strain reproduces by fission and contains a translocation between chromosomes 1 and 3 [1]. Though little is known about patterns of differential expression in planarian genes, we and others have previously identified microRNAs that are differentially expressed between the sexual and asexual strains [9], [10].

Neoblasts are somatic stem cells that give rise to all regenerated tissues in the animal and are sensitive to irradiation [11], [12]. Neoblast-enriched transcripts were traditionally identified by their decreased levels of expression following irradiation. Thus, characterization of the neoblast transcriptome will provide valuable insight into stem cell function and the regulatory mechanisms that control cellular differentiation. Large-scale RNAi screens have identified several genes that are important for neoblast function; moreover, phenotypes associated with RNAi knock-downs have been used to characterize the roles of specific genes in self-renewal and regeneration in adult somatic stem cell populations [13]. In addition, microarray analysis of gene expression changes upon irradiation have identified genes with important roles in neoblasts [14] while proteomic studies using 2D gel electrophoresis and mass spectrometry have helped characterize proteins expressed in planarian neoblasts [15].

Here, we have used RNA-Seq to characterize the *S. mediterranea* transcriptome. These experiments have revealed many uncharacterized genes, new alternatively spliced transcripts, and defined the expression profiles of these genes and transcripts in various strains and cell types. Comparison of the transcriptomes of intact and irradiated animals, in both the sexual and asexual strains, has allowed us to identify genes and transcripts that are expressed in a strain-specific or irradiation-sensitive manner. Moreover, transcriptome profiling of FACS purified neoblasts and differentiated cells revealed *S. mediterranea* neoblast-enriched transcripts. Strikingly, many of the neoblast-enriched genes are orthologs of genes whose expression is also enriched in human embryonic stem cells, suggesting that a core set of stem cell regulators are evolutionarily conserved across metazoan species.

## Results

### RNA-Seq Based de novo Transcript Assembly

To characterize the *S. mediterranea* transcriptome and to identify genes and mRNA isoforms that are expressed in a strain- and neoblast-specific manner, we performed single and paired-end RNA-Seq of mRNA isolated from sexual and asexual animals that were either non-irradiated or irradiated (GEO accession: GSE34326). RNA-Seq data generated from libraries prepared from *Smed-ago2(RNAi)*, *Smedwi-2(RNAi)* and *Smedwi-3(RNAi)* animals were also used to assemble transcripts (detailed results of the RNAi samples will be published elsewhere (D.Palakodeti and B.R.Graveley, in preparation)). In total, more than 67 million reads from these libraries mapped to the *S. mediterranea* genome (Table S1). We used Bowtie [16] and Tophat [17] to align the reads to the draft assembly of the *S. mediterranea* genome [3]. Cufflinks [18] was used for *de novo* assembly of transcript models using all reads. Subsequently, expression levels (fragments per kilobase of exon model per million mapped reads, FPKM) for all genes and transcript models were calculated for each sample individually.

Using a threshold of FPKM>1, which corresponds to transcripts with read densities in the 92nd percentile, we identified a total of 19,503 mRNA transcripts corresponding to 17,682 genes (Table 1, Dataset S1, Dataset S2). Our results are in close agreement with those from a recent transcriptome analysis in *S. mediterranea* which identified 22,698 isoforms from 17,628 genes [6]. Two additional reports estimate the number of *S. mediterranea* transcripts at 18,619 [8] and 25,053 [7]. In comparison, *Schistosoma mansoni* and *Schistosoma japonicum*, different species from the same genus, encode 11,809 [19] and 13,649 genes [20], respectively, and *S. japonicum* encodes 18,761 transcripts [20]. In contrast, the freshwater Cnidarian *Hydra magnipapillata*, which also undergoes regeneration, contains ∼20,000 protein coding genes [21]. *Caenorhabditis elegans, Drosophila melanogaster* and *Homo sapiens* encode 21,733, 15,512, and 22,287 genes, respectively [22], [23], [24].

**Table 1:** Summary of RNA-seq based genome annotation for *S. mediterranea*.

Annotation	Total
Genes	17,682
Exons	61,066
Introns	43,048
Transcripts (FPKM>1)	19,503
Transcripts with known RefSeq homologs (evalue≤1e-10)	9,343
Transcripts with Pfam domain annotation (evalue≤1e-3)	3,261
Transcripts with Smart domain annotation (evalue≤1e-3)	2,528

**Figure 1:**
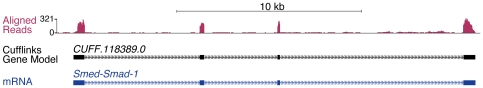
Validation of *S. mediterranea* transcript assemblies. Transcript assembly for *Smed-Smad-1 (CUFF.118389.0)*. *Smed-Smad-1* encodes 4 exons which are supported by RNA-Seq reads (maroon) and an *S. mediterranea* mRNA sequence.

A total of 78,333 EST and cDNA sequences have been generated from *S. mediterranea* [4], [5]. In addition, 3,412 full-length or partial mRNA sequences have been deposited at the NCBI. While assembly of our transcript models was not guided by these EST, cDNA or mRNA sequences, we used them to validate the assembled transcript models. EST, cDNA and mRNA sequences were aligned to transcript assemblies using MegaBLAST [25], [26]. For example, a gene model (*CUFF.118389*) encoding an ortholog of human Smad1 spans approximately 22 kb of genomic DNA, contains 4 exons and encodes one transcript (*CUFF.118389.0*) of 1.5 kb. Importantly, this gene model is fully supported and validated by an existing mRNA (NCBI accession: EF633692.1) (Figure 1). Overall, 45%, 29% and 9% of the transcripts, exons and introns we identified were supported by public ESTs or cDNAs, simultaneously validating many of our transcript models and indicating that we have identified many transcripts not represented in the public databases. Conversely, 64% of EST and cDNA sequences and 62% of mRNA sequences map to the assembled transcript models with an FPKM>1, again indicating that our dataset overlaps with the majority of the public ESTs and cDNAs. An additional 1% of EST and cDNA sequences map to transcripts with FPKM<1. On average, transcript models supported by ESTs have higher expression levels than transcript models without EST support (average FPKM of 43 and 8, respectively). The remaining public ESTs and cDNA sequences are either transcripts not detected in our data, or are derived from genomic DNA contamination. To further validate our transcript models, we performed RNA-Seq on mRNA isolated from the closely related species *Girardia tigrina*. Phylogenetic studies have shown that *S. mediterranea* and *G. tigrina* are both members of the family Dugesiidae [25], and that the maximum age of separation between the *Schmidtea* and *Girardia* genuses is 100 million years [26]. More than 23.5 million *G. tigrina* reads were used to assemble 14,514 transcript contigs (http://genome.vcell.uchc.edu/GenomeData02/Graveley_Lab_Public_Data/Planarian/G.tigrina_assembly.fasta) using Velvet [27]. Approximately 24% of the assembled *G. tigrina* transcripts could be mapped to the *S. mediterranea* transcript models. Twelve percent (12%) of *S. mediterranea* transcripts were supported by *G. tigrina* transcript assemblies. Thus, a total of 47% of transcript models and 46% of gene models derived from our *de novo* assemblies are validated by additional experimental support. Only transcripts with FPKM>1 were used for the remaining analyses.

Next, we examined the genomic features and sequence composition of the transcript models. The average *S. mediterranea* gene model is 6.9 kb and encodes a 931.2 bp transcript containing 3.1 exons. We determined that 132,792,101 bp (16.6%) of the assembled genome encodes detectably transcribed genes (including intronic regions) while 16,635,725 bp (2.7%) encodes transcribed exonic sequences. A total of 61,006 exons were identified, with an average length of 272.4 bp. We also identified 43,048 introns, with an average length of 2,668.2 bp. Planarian genes, exons, and introns were all longer than in *S. mansoni*, which have an average gene, exon, and intron lengths of 4.7 kb, 217 bp, and 1,692 bp, respectively [19]. The GC content of assembled transcripts was 36% (Table S2), in agreement with a recent study [7]. GC content of the *S. mediterranea* protein coding transcripts is also similar to that of the protein coding transcripts of *S. japonicum* (36%) [19] and *C. elegans* (36%) [28]. Taken together, the observed trends for GC content, exon and mRNA transcript lengths suggest that the 17,682 assembled genes represent a high confidence set of protein coding genes in the *S. mediterranea* genome.

### Annotation of Protein-Coding Transcript Models

To annotate the proteins encoded by the *S. mediterranea* transcripts, we aligned the assembled transcripts against curated protein coding and domain sequence databases. 48% (9,343) of *S. mediterranea* transcripts yielded high quality alignments (evalue≤1e^−10^) to known protein coding sequences (Table 1, Dataset S3) from one or more species, indicating that roughly half of assembled transcripts encode evolutionarily conserved coding sequences. These results are consistent with published estimates that 36%–62% of *S. mediterranea* transcripts are conserved in 1 or more species [6], [7], [8]. Overall, the number of *S. mediterranea* homologs is similar to *C. elegans* where 40% of protein-coding genes have homologs in other organisms [28]. Among *S. mediterranea* transcripts that have homologs, 32% were homologous to *S. mansoni*. Given our stringent alignment criteria, these results likely reflect the lower bound for the number of planarian genes with homologs. Of the transcript models without a clear ortholog in another species, 416 (4%) encode Pfam or Smart domains. Thus, in total, 50% of all *S. mediterranea* transcript models encode proteins with identifiable protein functions. This also indicates that approximately half of the *S. mediterranea* transcripts we identified potentially encode novel proteins. A recent study that examined the quantitative effect of tree-based genome selection on the pace of discovery of novel proteins and functions revealed that higher rates of novel protein family discovery were found in more phylogentically diverse taxa [29]. Thus, the *S. mediterranea* transcripts that lack homologs in mammals and insects may have homologs in more closely related species whose genomes have yet to be sequenced.

Protein domains were also mapped to *S. mediterranea* transcripts using RPS-BLAST [30], [31]. Domain hits with evalues≤1e^−3^ were accepted if ≥80% of the domain length mapped to the transcript sequence. Pfam and Smart domains were mapped to 3,261 and 2,528 isoform sequences, respectively (Table 1, Dataset S4). Domains were ranked by frequency of occurrence and we observed that the same types of domains were equally abundant among Pfam and Smart distributions. Analysis of the top 20 most abundant domains revealed that biological functions involving transcription, DNA/RNA-binding and signal transduction were the most frequently occurring Pfam domains (Table S3). Interestingly, 20% of the most frequently observed domains in *S. mediterranea* were among the top 20 most abundant domains in *C. elegans* [28]. These domains include the ankyrin repeat, RNA recognition motif (RRM), WD domain, helicase with conserved C-terminal domain, and the homeobox domain.

### Strain-specific Bias in Transcript Expression

**Figure 2:**
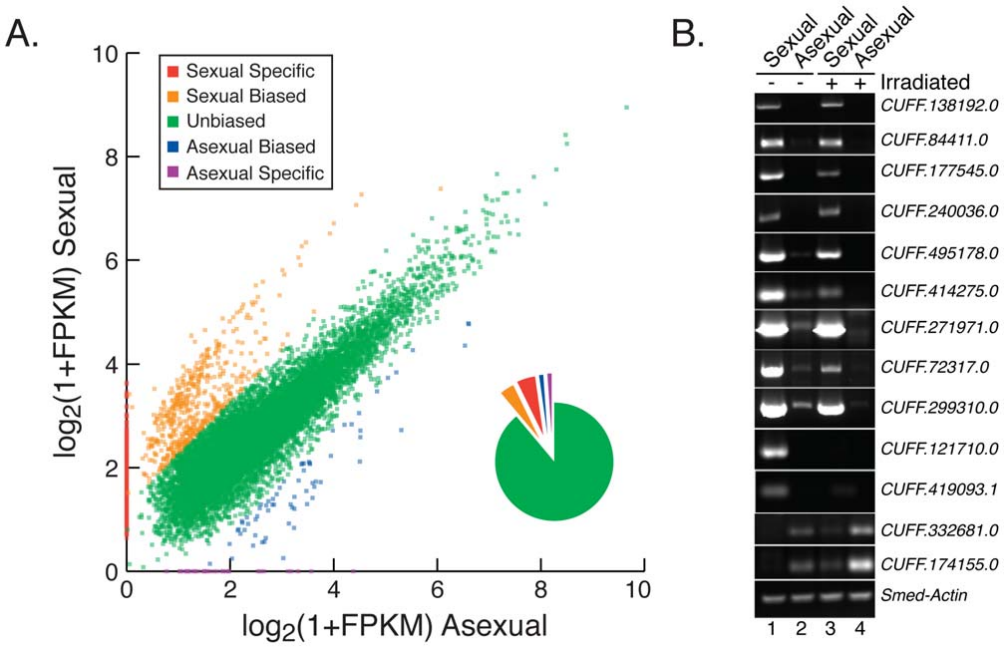
Strain-specific transcript expression. (A) Scatterplot of log_2_(1+FPKM) values for transcripts expressed in non-irradiated asexual (x-axis) and sexual (y-axis) animals. Biases in expression are categorized as follows: sexual-specific (red), sexual-biased (orange), unbiased (green), asexual-biased (blue) and asexual-specific (purple). The piechart (inset) shows the fraction of transcripts that fall into each category. (B) RT-PCR validation of strain-biased expression in sexual and asexual non-irradiated (−) and irradiated (+) samples; results are shown for 13 samples (*Smed-actin* used as a control).

We compared transcript expression profiles between sexual and asexual animals to determine whether strain-specific biases in expression can be observed, and if so, to what extent. The distribution of expression values is strongly correlated between sexual and asexual animals (r = 0.92; Pearson's correlation coefficient). Differentially expressed transcripts were identified by applying a fold-change threshold of 1.25, which allowed us to investigate a broad range of expression biases between the sexual and asexual strains. We grouped 12,088 transcripts that could be consistently classified (based on three independent cufflinks analyses) into one of the following categories: 1) asexual-specific, 2) asexual-biased, 3) unbiased, 4) sexual-biased, and 5) sexual-specific (Figure 2A; Dataset S9). Asexual-specific and sexual-specific transcripts are uniquely expressed in each strain. Transcripts designated as asexual-biased were expressed ≥1.25 fold higher in asexuals than sexuals, while those designated as sexual-biased were expressed ≥1.25 fold higher in sexuals than asexuals. Eighty-nine percent (89%) of transcripts displayed unbiased levels of expression (fold-change <1.25) (Figure 2A, green). In contrast, 11% of transcripts were expressed at least 1.25 fold higher in one strain than the other. Six percent (6%) were expressed specifically in either the sexual or asexual animals, while 5% of transcripts showed biased patterns of expression (Figure 2A). Strain-specific and biased patterns of expression were over 10 times more pronounced in the sexual strain where 1,159 transcripts were more abundantly expressed in the sexual strain compared to 98 transcripts that were expressed at higher levels in the asexual strain. RT-PCR results verified the strain specific and biased patterns of expression observed in the RNA-Seq data for the 13 transcripts shown in Figure 2B. These examples were selected because their gel images nicely illustrate the varying degrees of strain-specific expression depicted in Figure 2A. We compared the distribution of FPKM values between transcripts designated as differentially expressed and calculated signficant differences in expression for the sexual-biased (p = 5.6e-155; Mann-Whitney U test) and asexual-biased (p = 3.9e-12; Mann-Whitney U test) datasets.

We used Gene Ontology (GO) annotations to identify biological functions associated with differentially expressed transcripts. GO annotations were transferred from Pfam and Smart domains mapped to our transcripts. A limitation of this method is that transcripts without domain annotations are excluded from the GO analysis. On the other hand, transcripts are not excluded because they lack homology to other species, which was important for the functional analysis of the asexual strain, since asexually reproducing species are underrepresented in public sequence databases. A major difference between the two strains is that hermaphroditic, sexually reproducing animals undergo meiosis and genetic recombination, while asexual animals reproduce by fission. Strain-specific or biased transcripts were enriched for genes that reflect the reproductive mode of the strains. For example, we compared the frequency of Gene Ontology annotations between transcripts that showed sexual-biased versus unbiased expression, and found that genes involved in protein polymerization, GTPase activity and GTP binding were significantly enriched in transcripts that exhibited sexual-bias in expression (Table 2; p<0.00001; Chi-square test). Proteins with GTPase and protein polymerization activity are associated with meiotic spindle assembly [32] and similar functional enrichment was previously observed in sexually mature females and males in *Daphnia pulex* [33]. GTPase activity has been linked to meiotic spindle assembly, which is required for gametogenesis [34]. We performed a similar analysis between transcripts that showed asexual-biased versus unbiased expression and found that genes involved in homophilic cell adhesion and calcium ion binding were significantly enriched in transcripts that exhibited asexual-bias in expression (Table 2; p<0.0005; Chi-square test). Interestingly, asexual reproduction in tunicates is dependent upon a budding mechanism facilitated by calcium-dependent cell adhesion molecules including lectins and cadherins, which facilitate budding through cell aggregation and migration [35]. Among transcripts classified as asexual-specific or asexual-biased in our dataset, we identified several cell adhesion genes, which are known to play roles in asymmetrical cell division [36], [37], a process similar to budding.

**Table 2:** Strain-specific patterns of GO category enrichment.

	Sexual-biased (n = 782)	Unbiased (n = 2,758)	P-value
Protein polymerization	52	4	1.28E-139
Protein complex	52	4	1.28E-139
GTPase activity	33	20	4.24E-24
GTP binding	44	85	1.76E-6

	Asexual-biased (n = 207)	Unbiased (n = 1,974)	P-value
Homophilic cell adhesion	15	25	3.74E-18
Calcium ion binding	16	34	1.85E-15
Membrane	28	114	4.33E-11
Ion transport	6	12	4.99E-7
Ion channel activity	5	11	1.29E-5
Sequence-specific DNA binding	12	56	0.0002
Transcription factor activity	13	66	0.0005

GO category counts were obtained from Pfam domain annotations. Enrichment was determined by comparing category counts between transcripts. Enrichment in sexual animals was determined by comparing category counts between the sexual-specific/sexual-biased and unbiased categories; enrichment in asexual animals was determined by comparing the asexual-specific/asexual-biased and unbiased categories (transcripts that showed unbiased expression were used as the control set). The test for significance was performed using the Chi-square test. P-values were adjusted using the Bonferroni correction.

### Discovery of Alternative Splicing Events

**Figure 3:**
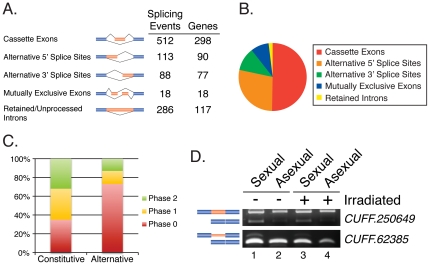
Classification of alternative splicing events. (A) Number of splicing events and genes detected for 5 classes of alternative splicing events: cassette exons, alternative 5′ splice sites, alternative 3′ splice sites, mutually exclusive exons and retained introns. (B) Percentage of each class of alternative splicing event shown in piechart. (C) Frame preservation in constitutively and alternatively spliced cassette exons; phase 2 and phase 1 exons are out-of-frame; phase 0 exons are in-frame. (D) RT-PCR validation of strain-specific alternative splicing events in non-irradiated (−) and irradiated (+) sexual and asexual animals. A cassette exon (*CUFF.250649*) is shown in top gel, while a retained intron (*CUFF.62385*) is shown in the bottom gel.

Although alternative splicing is known to play important roles in increasing protein diversity and regulating gene expression in most eukaryotes [38], the role of alternative splicing in planarians is unexplored. We identified 1,435 genes (9%) with multiple transcript models; 80% of these genes have only two transcript models, while the remaining 20% have more than two. To identify alternative splicing events among these 1,435 genes, as opposed to alternative transcription initiation or polyadenylation sites, we analyzed the transcript models for cases of cassette exons, retained introns, and alternative 5′ and 3′ splice sites. This analysis revealed 512 cassette exons in 298 genes, 286 intron retention events in 117 genes, 36 mutually exclusive exons in 18 genes, 113 alternative 5′ splice sites in 90 genes, and 88 alternative 3′ splice sites in 77 genes, for a total of 1,035 alternative splicing events in 563 genes (Figures 3A and 3B). In total we identified alternative splicing events in 3.2% of *S. mediterranea* genes.

We catalogued the effects of alternative splicing on protein reading frame by classifying alternatively spliced cassette exons as either inframe or frameshift events. Alternatively spliced exons with lengths divisible by three were classified as inframe, while exon lengths not divisible by three were classified as frameshift events. This analysis revealed that 73% of cassette exons preserved reading frame, while 27% resulted in frameshift (Figure 3C). In contrast, 35% of constitutively spliced exons preserved reading frame, while 65% resulted in frameshift. Thus, alternatively spliced cassette exons show a strong bias toward frame preservation that is not observed in constitutive exons (p = 3.78×10^−13^; Chi-square test). Our results are consistent with trends in frame bias that have been reported for alternatively spliced cassette exons in many organisms including *Drosophila* [23] and human [39], [40], [41]. These data suggest that the majority of cassette exons are under selective pressure to preserve reading frame. We observed no significant differences in frame preservation between retained (39% resulted in frame preservation) and constitutively spliced (32% preserved frame) introns (p = 0.155; Chi-square test). The frame preservation characteristics of these exons are consistent with their classification as constitutive and cassette exons and suggest that authentic transcript models have been generated from our methods.

### Strain-specific Alternative Splicing

Sex-specific alternative splicing is well known in *Drosophila* [23], [42] and primates [43]. We identified several strain-biased alternative splicing events in the RNA-Seq data from sexual and asexual strains. For example, *CUFF.250649* encodes a Muscleblind-like protein and contains a cassette exon in the coding region. The cassette exon of *CUFF.250649* is included in both sexual and asexual animals, but is occasionally skipped in sexual animals (Figure 3D). The cassette exon is frame preserving (333 bp; 111 amino acids) and encodes a serine/threonine-rich region comprised of putative phosphorylation sites. Muscleblind-like genes encode a family of evolutionarily conserved RNA-binding proteins that regulate alternative splicing [44], [45], [46].

We also identified a retained intron in *CUFF.62385* which encodes a protein with close homology to the ATP-transporter Haf-6. Expression studies in *C. elegans* showed that Haf-6 is required for RNAi and is expressed predominantly in intestine and germline [47]. The retained intron is 29 bp and appears to be located in the 5′ UTR, indicating a possible role in regulating mRNA stability and/or translation. The intron is retained in sexual animals (Figure 3D).

Taken together, these results demonstrate that regulated alternative splicing contributes to transcript diversity in *S. mediterranea*.

### Identification of Neoblast-enriched Transcripts

Adult planarians possess a population of pluripotent stem cells called neoblasts that regulate regeneration following injury. Transcripts involved in regeneration and stem cell self-renewal pathways have been shown to decrease following irradiation which depletes the neoblasts [14], [48], [49]. Neoblast-enriched transcripts have therefore traditionally been identified by comparing expression levels before and after irradiation; transcripts that exhibit decreased levels of expression upon irradiation in sexual and asexual strains are classified as neoblast-enriched. Starting with a set of 17,084 transcripts, we identified 4,726 irradiation-sensitive transcripts (28%) that showed decreased expression in both strains following irradiation and 12,358 irradiation-insensitive transcripts (72%) whose expression levels did not decrease (Dataset S5). Transcripts were designated irradiation-sensitive if their expression levels showed a decrease in sexual and asexual strains following irradiation (i.e., expression is lower in sexual and asexual irradiated samples when compared to sexual and asexual non-irradiated samples). A previous study examined the effects of irradiation on transcripts from the asexual strain, and classified ∼2,300 as irradiation-sensitive [6]. Thirty-three percent (33%) of the asexual irradiation-sensitive transcripts identified by Blythe et al [6] were also classified as irradiation-sensitive in our analysis.

**Figure 4:**
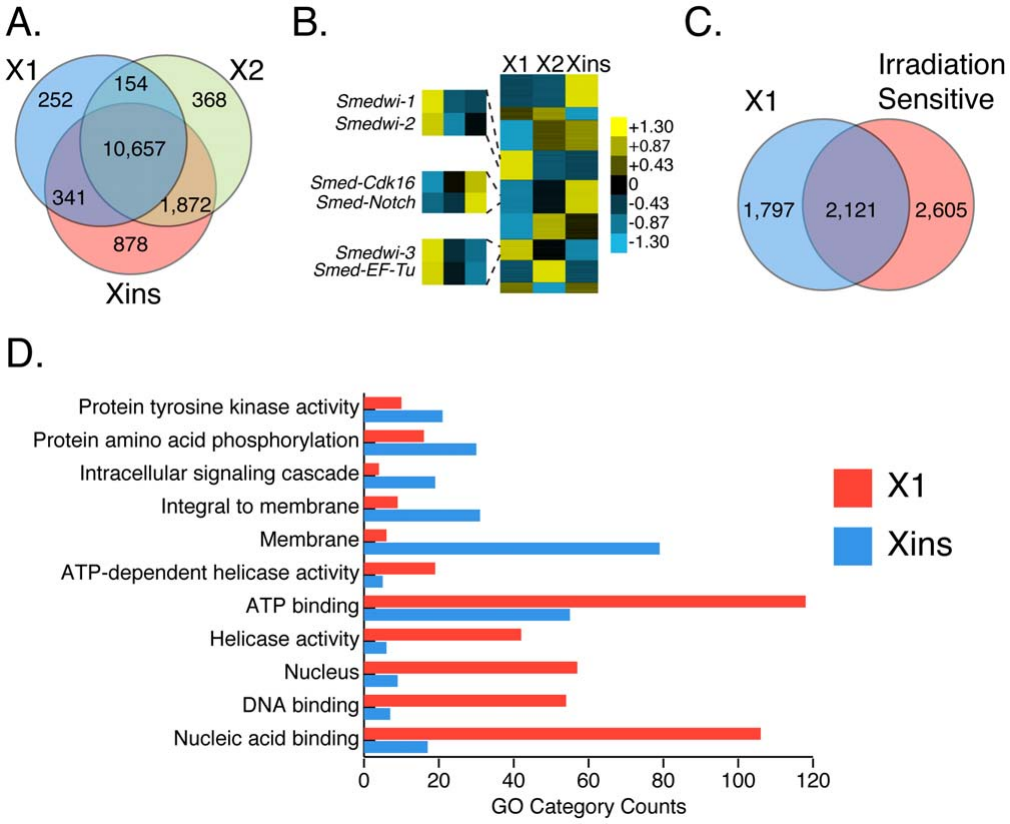
Transcript expression profile of *S. mediterranea* neoblasts. (A) Overlap in transcript expression between the X1 (blue), X2 (green) and Xins (red) cell populations displayed as Venn Diagrams. (B) Expression values (FPKM) for transcripts in the X1 (column 1), X2 (column 2) and Xins (column 3) cell populations (expressed as *Z*-scores) are shown in the heat map. *Z*-score colors range from yellow (above the mean) and blue (below the mean). (C) Overlap in expression between transcripts enriched in FACS generated X1 cells and transcripts classified as irradiation-sensitive in sexual and asexual strains. (D) GO category counts (x-axis) for 11 categories (y-axis) associated with transcripts enriched in X1 (blue) and Xins (red) cells. Category counts are signficantly different between X1 and Xins populations for all categories (p<0.0009; Chi-square). P-values were adjusted using the Bonferroni correction.

To verify that irradiation-sensitive transcripts are indeed neoblast-enriched, we performed RNA-Seq on fluorescence-activated cell sorting (FACS) purified neoblasts and differentiated cells. Cells from sexual animals were sorted into 3 distinct populations according to DNA content and cell size as described previously [9], [50]: 1) irradiation-sensitive proliferating pluripotent neoblasts (X1), 2) irradiation-sensitive replicating neoblasts in G_1_ phase (X2), and 3) irradiation-insensitive differentiated cells (Xins). Significant reductions in the X1 and X2 populations were observed following irradiation in both sexual and asexual strains (Figure S1). We observed a 6-fold and 4-fold decrease in the X1 population in sexual and asexual strains, respectively, and a 9-fold and 5-fold reduction in the X2 population in sexual and asexual strains, respectively. We generated approximately 25 million reads each from the X1, X2 and Xins libraries (∼75 million total) from the sexual strain and used the data to analyze expression patterns between the populations. Reads generated from FACS-sorted libraries were mapped to the previously derived transcript coordinates. A total of 14,522 transcripts were expressed in one or more cell populations (11,404 transcripts were expressed in X1, 13,051 in X2, and 13,748 in Xins) and 10,657 transcripts (73%) were expressed in all cell populations, albeit at different levels (Figure 4A). Cell-specific transcript expression was observed in all cell types: 252 transcripts were specifically expressed in X1 neoblasts, while 368 and 878 transcripts were specifically expressed in X2 and Xins cells, respectively (Figure 4A). Next, we classified transcripts according to the cell type in which they are most predominantly expressed. Transcripts were classified as X1-enriched if expression in X1 cells was greater than expression in the X2 and Xins populations; likewise, X2-enriched transcripts were more highly expressed in X2 than in X1 and Xins, and Xins-enriched transcripts were more highly expressed in Xins than in X1 and X2. Seventeen percent (17%) of transcripts (3,429 transcripts; 3,311 genes) were most highly expressed in the proliferating X1 neoblast population and showed lower levels of expression in X2 and Xins cell populations (Table 3). In comparison, 17% (3,276 transcripts; 3,180 genes) and 23% (4,525 transcripts; 4,370 genes) of transcripts were found to be most highly epxressed in X2 and Xins populations, respectively (Table 3). We used multiple tests to confirm that our classification schema is statistically valid. Analysis of variance revealed significant differences between all 3 datasets (ANOVA; p<0.0001). Pairwise comparisons yielded significant results for the X1 vs X2 (p = 0.0001;Student's ttest) and X1 vs Xins (p = 2.9e-06; Student's ttest) comparisons, but not the X2 vs Xins (p = 0.258; Student's ttest) comparison. Similar results for pairwise comparisons were obtained using Tukey's HSD test: significant differences were observed for X1 vs X2 (p<0.01) and X1 vs Xins (p<0.01), but not the X2 vs Xins comparison.

**Table 3:** Number of genes and transcripts enriched in *S.mediterranea* and Human cell populations.

	*S. mediterranea*			Human	
	X1	X2	Xins	hESCs	Day-6 EBs
Genes	3,311	3,180	4,370	3,475	4,546
Transcripts	3,429	3,276	4,525	4,235	5,681

*S. mediterranea* adult cells include the X1, X2 and Xins populations and human cells include embryonic stem cells (hESCs) and day-6 embryoid bodies (hEBs). *S. mediterranea* X1 neoblasts and hESCs are both pluripotent, irradiation-sensitive cell populations.

We compared transcript expression profiles across all 3 cell types by standardizing and converting the raw expression values into Z-scores and displaying the data as a heat map (Figure 4B, Dataset S6). Genes enriched in the X1 neoblast population included *Smedwi-1* (*CUFF.400999.0*), *Smedwi-2* (*CUFF.92311.0*), *Smedwi-3* (*CUFF.190314.0*), and *EF-TU* (*elongation factor Tu (CUFF.346863.0)*) (Figure 4B). *Smedwi-1, -2, and -3*, and *Smed-EF-Tu* were previously shown to be enriched in pluripotent neoblast cells [14], [48], [49]. Examples of genes whose expression is most pronounced in differentiated Xins cells include those with homology to *S. mansoni Notch* (*Smed-Notch*; CUFF.160960.0) and *Xenopus Cdk16* (*Cyclin-dependent kinase 16* (*Smed-cdk16*; CUFF.149668.0)) (Figure 4B). Studies have shown that *Notch* is an important cell signaling molecule that controls developmental cell fate and differentiation in several cell lineages [51], [52]. *Cdk16* (also known as *Pctaire1 kinase*) is also important for differentiation as it is predominantly expressed in terminally differentiated tissues such as neurons and testes [53], [54].

To validate the use of irradiation sensitivity as a measure of neoblast expression, we compared the transcripts that were predominantly expressed in X1 cells and those that were irradiation-sensitive in both sexual and asexual animals. We identified 2,121 transcripts that were both enriched in X1 cells and sensitive to irradiation in both strains (Figure 4C). However, 1,797 X1-enriched transcripts were not irradiation sensitive and 2,605 irradiation-sensitive transcripts were not enriched in X1 cells. These data indicate that while there is agreement between irradiation-sensitive transcripts and neoblast-enriched transcripts, not all irradiation-sensitive transcripts are enriched in neoblasts and not all neoblast enriched transcripts are irradiation-sensitive. We then compared the overlap between transcripts classified as irradiation-sensitive in sexual and asexual strains with those predominantly expressed in the X1, X2 and Xins cell populations. Our analysis revealed that 54% of transcripts predominantly expressed in the X1 population are also classified as irradiation-sensitive in sexual and asexual animals, as measured by a decrease in expression in both strains following irradiation. Likewise, 16% and 17% of transcripts predominantly expressed in the X2 and Xins populations, respectively, are also classified as irradiation-sensitive in sexual and asexual animals. Thus, as expected, a substantially larger fraction of irradiation-sensitive transcripts show overlap with the X1 population than the X2 and Xins populations.

To examine the functional implications of the observed gene expression patterns, we performed GO analysis of the enriched sets of transcripts and genes. GO annotations were transferred from the Pfam and Smart domains mapped to our transcripts. Comparison of GO categories between transcripts predominantly expressed in X1 neoblasts versus those enriched in Xins differentiated cells revealed significant and opposing patterns of enrichment (Figure 4D) (p<0.0006; Chi-square test). GO categories such as nucleic acid binding, DNA-binding, helicase activity, ATP binding, nucleus and regulation of transcription were enriched in X1 neoblasts and depleted in Xins cells. Likewise, GO categories such as protein binding, protein amino acid phosphorylation, oxidation reduction, protein kinase activity and metabolic process were enriched in Xins differentiated cells and depleted in X1 neoblast. These data indicate that pluripotent and differentiated planarian cells express genes with unique and opposing functional signatures.

### Conserved Transcripts Expressed in Planarian and Human Stem Cells

Given the functional similarities between planarian neoblasts and human embryonic stem cells, we hypothesized that humans and planarians might share a core set of genes required for stem cell maintenance and self-renewal. We therefore examined whether the *S. mediterranea* neoblast-enriched transcripts corresponded to transcripts enriched in human embryonic stem cells. To do this, we identified *S. mediterranea* genes with human orthologs and then identified orthologous transcripts expressed in pluripotent and differentiated cell populations from both species. Using a conservative reciprocal best hit methodology, we identified 1,729 putative orthologous gene pairs, or approximately 10% and 8% of *S. mediterranea* and human genes, respectively (Dataset S7). The *S. mediterranea*-human orthologs are involved in a variety of important biological processes, including protein amino acid phosphorylation (31%), transmembrane transport (19%), regulation of transcription (19%), signal transduction (19%), oxidation reduction (15%), proteolysis (8%) and cell cycle regulation (8%). Genes that regulate DNA repair, DNA replication, chromatin modification, translation and mRNA processing were also present in the set of orthologous genes.

We searched for evidence of orthologous transcript expression profiles in *S. mediterranea* and human stem cell populations. We first determined the number of *S. mediterranea* genes enriched in the X1, X2 and Xins cell populations that have a human ortholog. Starting with the 1,729 *S. mediterranea*-human orthologous gene pairs identified in our study, we further classified orthologs according to the cell population (X1, X2 or Xins) where they are most highly expressed. We determined that 74% of orthologous gene pairs were detected in at least one cell population; 26% of orthologs were most highly expressed in the X1 population, 25% were most highly expressed in the X2 population, and 23% were most highly expressed in the Xins population.

RNA-Seq data generated from undifferentiated H9 hES cells and H9-derived day-6 human embryoid bodies (hEBs) was used for the analysis. As embryoid bodies have begun the process of differentiation, human stemness genes enriched in undifferentiated hESCs show a pattern of decreased expression in differentiated EBs. Expression values were generated for transcripts expressed in hESCs and day-6 EBs (Dataset S8). A detailed analysis describing this and other human RNA-Seq datasets will be published elsewhere (M.Horowitz, M.O.Duff, X.J.Li and B.R.Graveley, in preparation). Briefly, human transcripts were classified as stem cell-enriched if their expression level in hESCs was at least 1.3 fold greater than the corresponding expression level in hEBs. Our results identified 4,235 transcripts (3,475 genes) enriched in hESCs and 5,681 transcripts (4,546 genes) enriched in day-6 EBs (Table 3). A fold-change threshold of 1.3 has been used in microarray experiments [55] and resulted in statistically significant results when used to identify hESC-enriched and hEB-enriched transcripts in our analysis (p = 2.6e-4 for hESC-enriched; p = 4.6e-4 for hEB-enriched; Mann-Whitney U test).

Next, we compared expression profiles between pluripotent and differentiated cells in *S. mediterranea* and human to identify orthologous genes whose expression is also conserved. Our analysis revealed that 13% (455/3,429) of the genes predominantly expressed in X1 neoblasts and 8.7% (397/4,525) of the genes predominantly expressed in differentiated Xins cells have human orthologs. We then determined the fraction of ortholgous genes in the X1 and Xins populations whose expression is also conserved in hESCs and hEBs. After normalizing for total number of orthologous gene pairs, we found that expression of 95 genes was conserved in X1 neoblasts and hESCs (Table S4), while 123 genes were conserved in Xins and hEB populations (Table S5). We detected a significant difference between the proportion of orthologous genes conserved in the X1-hESC (95/455) and Xins-hEB (123/397) populations (p = 0.0007; Z-ratio), indicating that ortholog expression in X1-hES cells is slightly less conserved than in Xins-hEB populations. These results suggest that, at least among *S. mediterranea*-human orthologs, the expression of genes that control differentiation is slightly more conserved than the expression of genes that maintain stem cell pluripotency.

Sixty-four percent (64%) (61/95) of the *S. mediterranea*-human stem cell enriched orthologs were classified as neoblast-enriched in planarians in both X1 neoblast cells and by irradiation-sensitivity expression profiles in both sexual and asexual strains (i.e., overlap between X1 and irradiation-sensitive sets in Figure 4C), indicating that the majority of planarian conserved stemness genes are classified as neoblast-enriched using separate classification methods. Functional annotations for these conserved sets are consistent with their biological activity: the function of genes with conserved expression in X1/hESCs included regulation of transcription, chromatin modification, cell division and positive regulation of cell proliferation, while the function of genes conserved in Xins/hEBs included protein amino acid phosphorylation, regulation of transcription, transmembrane transport, signal transduction and cell differentiation. These data indicate that the expression of a common set of genes is enriched in pluripotent and differentiated stem cell populations from planarians and humans, and that planarians are useful model organisms for studying stem cell biology.

## Discussion

Our analysis of transcript expression in different strains and cell types of *S. mediterranea* has produced sequence data, functional annotation, expression profiles and candidate gene sets for thousands of *S. mediterranea* transcripts. Comparison of our transcript models with publicly available *S. mediterranea* EST/cDNA and mRNA sequences provides independent experimental support for 49% of our computationally derived models. Furthermore, 50% of our transcript models map to known homologs and protein domains. In total, we identified 19,503 coding transcripts, consistent with other recently published reports [6], [7], [8]. Although transcriptome assemblies for *S. mediterranea* have been previously published [6], [7], [8], we also examined expression dynamics between sexual and asexual animals, upon irradiation, and in specific purified cell populations. Moreovoer, we have identified a core set of evolutionarily conserved genes whose expression is enriched in both *S. mediterranea* and human stem cells. Thus, these results have tremendous implications for stem cell biology.

### Strain-specific Expression

A striking finding of our analysis is that ∼1,200 transcripts are differentially expressed between the sexual and asexual strains (Figure 2A), the majority of which show a strong bias toward sexual-specific expression. These results are consistent with earlier findings that planarian small RNAs exhibit differential patterns of expression in sexual and asexual strains [10], [49]. Using RT-PCR, we verified the strain-specific expression of genes likely to play roles in sexual reproduction, and found that the gene encoding a homolog of *Vcp/p97 (valosin-containing protein/p97 (CUFF.138192.0))*, which has been implicated in sexual reproduction by way of sperm-attracting activity in ascidians [56], is expressed specifically in sexual animals (Figure 2B). We also found that *Smed-hnRNPA2 (CUFF.177545.0)*, which encodes a homolog of hnRNPA2, and *Smed-rap55 (CUFF.414275.0)*, which encodes a RAP55 homolog, are much more strongly expressed in sexual animals than asexual animals (Figure 2B). A recent study showed that RNAi depletion of *Smed-hnRNPA2* and *Smed-rap55* resulted in defects in spermatid elongation, indicating that these genes play important roles in male germ cell development and spermatogenesis [57]. Thus, the RNA-seq data used in this analysis has been useful in identifying novel candidates in germline development (*Vcp/p97*), and for generating transcript expression patterns that are consistent with previously published reports (*Smed-hnRNPA2* and *Smed-rap55*). In addition, genomewide analysis of alternative splicing revealed many transcripts that are alternatively spliced in a strain-specific manner. It is likely that further study of the set of strain-specific transcripts and alternative splicing events identified in this study will provide insight into both germline development (sexual-specific transcripts) and the mechanism of reproductive fission (asexual-specific transcripts).

### Alternative splicing in S. mediterranea

Our RNA-Seq data allowed us to identify over 1,000 alternative splicing events in *S. mediterranea* (Figure 3A). The observation that alternatively spliced cassette exons tend to be frame-preserving (Figure 3C) is consistent with known properties of cassette exons in other organisms [23], [39], [40], [41]. Interestingly, several of the alternative splicing events we identified are strain-specific such that sexual and asexual animals express different isoforms (Figure 3D). We predict that many of the splicing events we identified are also expressed in a tissue or cell-type specific manner, though further experiments are required to explore this possibility.

### Biological Roles of S. mediterranea Stemness Genes

Our analysis revealed that 17% of transcripts are irradiation sensitive and expressed in X1 cells and are therefore candidates for stemness genes. Several of these genes were involved in biological pathways that regulate chromatin remodeling, cell cycle progression, apoptosis, and regulation of cellular proliferation and cell fate determination. Genes involved in chromatin remodeling are thought to play important roles in maintaining embryonic stem cell self-renewal and pluripotency [58]. It has been hypothesized that DNA damage, a major cause of age-dependent stem cell decline, is tightly regulated in stem cells via maintenance of pathways such as nucleotide excision repair and telomere maintenance [59]. We identified several neoblast-enriched genes that are implicated in DNA repair, including the DNA mismatch repair protein Msh2, which was recently functionally characterized in *S. mediterranea* [60].

### Evolution of stemness genes in multicelluar organisms

We identified nearly 100 evolutionarily conserved genes whose expression patterns are also conserved in planarian X1 neoblasts and human ESCs. These genes are involved in chromatin remodeling and regulation of transcription, cell cycle function, apoptosis and cell proliferation pathways. These biological processes are widely known to control stem cell maintenance and self-renewal in pluripotent stem cell populations in human [58], [61], [62]. For example, this set of genes includes the transcriptional silencers *Bcl11a* and *Sirt1* and the mitotic kinase *Aurora A*. *Bcl11a* has been implicated in the maintenance of stem cell self-renewal in humans [63], while *Sirt1* regulates the expression of specific developmental genes in pluripotent hESCs [64]. *Aurora A*, a mitotic kinase with oncogenic potential, is highly expressed in hESCs and plays an important role in chromosomal stability [65].

Strikingly, orthologs of the mammalian genes *Myc*, *Nanog*, *Klf4*, *Oct4 (Pou5f1)* and *Sox2*, which induce pluripotency of differentiated somatic cells [66], [67], do not appear to be present in the draft assembly of the *S. mediterranea* genome. However, all five genes were expressed in the hES and hEB cell populations we analyzed. A similar analysis performed in *Hydra magnipapillata*, a species also capable of regeneration, showed that homologs of *Nanog*, *Klf4* or *Oct4* were not present in the *Hydra* genome [21]. Homologs of *Myc* and *Sox2* were identified in *Hydra*, but the evolutionary relationship between the homologs was not clear [21]. The authors concluded that the pluripotent stem cells in *Hydra* may have arisen independently from mammalian stem cells. This theory might explain the absence of these genes in *S. mediterranea*. The KLF, OCT and SOX transcription factor families contain multiple paralogs, so it is possible that gene duplication events played a role in the evolution of lineage-specific pluripotent stem cell lines. We identified paralogs of these genes that were enriched in the *S. mediterranea* X1 neoblast population; genes in this set include *Klf1*, *Pou4*, *Pou6f1*, *Sox14* and *Sox3*. Members of these genes families have been linked to early development [68], [69], [70], [71], [72]. It is plausible that paralog expansion within these gene families led to the acquisition of novel functions [69], [73], such that paralogous genes involved in stem cell maintenance provided functional redundancy, or led to species-specific variation of function within genes unique to planarian development.

## Materials and Methods

### Experimental Procedures

#### Planarian cultures

Sexual [4] and asexual [5] strains of *S. mediterranea* were provided by P. Newmark (University of Illinois) and A. Sanchez Alvarado (University of Utah/HHMI). Animals were maintained at 22°C in ddH_2_O supplemented with 1.6 mM NaCl, 1.0 mM CaCl2, 1.0 mM MgSO4, 0.1 mM MgCl2, 0.1 mM KCl, 1.2 mM NaHCO3 and fed homogenized organic beef liver as previously described [74]. All animals were starved 1 week prior to experiments.

#### mRNA-Seq library preparation

Total RNA was isolated from sexual and asexual strains of *S. mediterranea* before and after irradiation using Trizol. Total RNA (5 ug) was used to prepare the transcriptome library using the mRNA-Seq sample library preparation kit (Illumina) following the manufacturers instructions. Briefly, mRNA was purified using magnetic oligo-dT beads and fragmented. First strand synthesis of cDNA was done using SuperscriptII reverse transcriptase followed by RNase H treatment and second strand cDNA synthesis was performed using DNAPol1. Paired end adaptors (Illumina) were ligated to 5′ and 3′ ends of DNA fragments followed by PCR amplification using adaptor specific primers. PCR amplified products of 300 bp were gel purified, quantitated by Nanodrop and Bioanalyzer and sequenced on an Illumina GAIIx.

Biological replicates of RNA-Seq libraries were not used in this analysis. RNA was collected and pooled from multiple specimens. A total of 40 worms were used for RNA-Seq library preparation (10 each for sexual and asexual non-irradiated and 10 each for sexual and asexual irradiated libraries). The FACS analysis used a total of 80 sexual worms for 4 rounds of sorting (20 worms for each round).

RNA-Seq of undifferentiated H9 human embryonic stem cells (hESCs) and differentiated day-6 human embryoid bodies (hEBs) will be described elsewhere (M.Horowitz, M.O.Duff, X.J.Li, and B.R.Graveley, in preparation).

#### RNAi

RNAi depletion of *Smed-Ago*, *Smedwi-2* and *Smedwi-3* was performed as described previously [49].

#### gamma-Irradidation

Sexual and asexual animals were gamma-irradiated with a cesium source with 90 Gy treatments as previously described [49]. FACS analysis was performed 3 days after irradiation.

#### Fluorescence-activated cell sorting analysis

FACS analysis was carried out as previously described [48], [49], [50]. Briefly, planarians were cut into small pieces with a sterile scalpel on ice in cold calcium, magnesium-free medium (CMF) (15 mM HEPES, 400 mg/L NaH_2_PO_4_, 800 mg/L NaCl, 1200 mg/L KCl, 800 mg/L NaHCO_3_, 240 mg/L glucose at pH 7.3) and then washed twice with CMF. The fragments were then soaked in CMF containing 0.25% (w/v) Trypsin (GIBCO) and 1% BSA (Fisher Scientific) at 4°C for 18 hours to maximize penetration of the enzyme with little trypsin activity. The fragments were then rocked for 20 minutes at 20°C and completely dissociated into single cells by gentle pipetting. The cell mixture was then sequentially filtered through 40-µm and 20-µm nylon filters (Millipore). Cells were collected by centrifugation, resuspended in fresh CMF supplemented with 1% BSA and 0.5 mg/mL Calcein AM (Sigma), and incubated at room temperature for 20 minutes. Cells were collected and resuspended in CMF supplemented with 1% BSA, 18 mg/mL Hoechst 3342 (Sigma), and 2 mg/mL propidium iodide (Sigma). The samples were then analyzed with a LSR II flow cytometer (Becton-Dickinson). A total of 80 sexual worms were used for 4 rounds of sorting (20 worms for each round).

#### Semiquantitative RT-PCR

First strand cDNA was synthesized from 1 µg of total RNA using SuperScriptII (Invitrogen). PCR amplification was performed for 20, 25 and 30 cycles and the samples resolved on 1.2% agarose stained with ethidium bromide. The primers used for this study are included in Table S6.

#### Experimental Validation of Predicted Transcripts

Experimental validation of transcript models and predicted patterns of expression was performed using semi-quantitative RT-PCR. We selected a total of 34 transcripts as candidates for RT-PCR and constructed PCR primers against their sequences. Transcripts predicted to be differentially expressed or alternatively spliced were preferentially chosen as candidates for validation, and when appropriate, we chose candidates that were highly expressed or biologically interesting. Validation studies were designed to test the expression of 1) transcript assemblies, 2) differential expression, and 3) alternative splicing. Our results showed that 94% (32/34) of predicted transcripts were experimentally validated based on the production of a PCR product of the correct size, confirming that our transcript assembly methods were successful (2 candidates failed to produce PCR products). In addition, a validation rate of 80% (21/26) was estimated for predicted patterns of differential expression, confirming that FPKM values are a reasonable estimate of endogenous transcript expression. The PCR results for the 13 transcripts shown in Figure 2B are included in this set. Finally, a validation rate of 75% (6/8) was estimated for predicted alternative splicing events on the basis that candidates showed multiple PCR products of different sizes that corresponded with the predicted transcript sizes. Primer sequences and gel results for the 32 candidates validated by RT-PCR are included in Table S6 and Figure S2.

#### mRNA-Seq based transcript assembly

The *S. mediterranea* draft genome assembly (version 3.1) consists of ∼900 Mb distributed over 43,294 supercontigs (http://genome.wustl.edu/genomes/view/schmidtea_mediterranea/). To facilitate the analysis the supercontigs were sorted by decreasing size and concatenated into nine pseudochromosomes of ∼100 Mbp each with 100 ‘N’ residues separating each contig (http://genome.vcell.uchc.edu/GenomeData02/Graveley_Lab_Public_Data/Planarian/S.mediterranea_pseudochromosomes.fasta). All alignments were performed using the nine pseudochromosomes. This method was used to reduce the complexity of the reference genome and does not introduce split genes, as there are no scaffolds for which to align the supercontigs. The supercontigs are the largest scaffolds available. Transcripts were assembled using Bowtie-0.12.1 [16], TopHat-1.1.1 [17], and Cufflinks-0.8.1 [18]; default parameters were used.

Different methods of *de novo* transcript assembly were used for *S. mediterranea* and *G. tigrina*, because the reference genome for *G. tigrina* is not available. A total of 23,532,916 *G. tigrina* reads of 40 bp were used to assemble 14,514 transcript contigs with Velvet 0.3 [27] using *k* = 15. This resulted in contigs with an average length of 140 bp and an average coverage of 8.7×.

#### Functional annotation

*S. mediterranea* EST/cDNA and mRNA sequences were aligned to assembled transcripts using MegaBLAST. Homologs were identified by aligning RefProt protein sequences against translated mRNA transcripts using BLASTP with an e-value threshold of 1e^−10^. Pfam and Smart domains were mapped to assembled transcripts using RPS-BLAST with an e-value threshold of 1e^−3^. Gene Ontology (GO) annotations were used to assign biological functions to genes included in this study [75]. GO annotations were transferred from Pfam and Smart annotations mapped to our transcripts.

#### Expression profiles

Raw expression values (FPKM) for sexual and asexual non-irradiated and irradiated samples and FACS cell populations were standardized and converted into *Z*-scores. *Z*-scores were clustered using Cluster 3.0 [76] and displayed as heat maps using Java TreeView [77].

## Supporting Information

Dataset S1 **Fasta file of** ***S. mediterranea*** **assembled transcript sequences with FPKM>1.** Each header line contains a unique cufflinks transcript id.
(ZIP)

Dataset S2 **Excel spreadsheet containing information about relative transcript abundance levels (FPKM), absolute depth of read coverage across the transcript (coverage), and transcript length (bp) for assembled transcripts with FPKM>1.**
(XLS)

Dataset S3 **Excel spreadsheet of** ***S. mediterranea*** **homologs.** Homologous protein sequences were identified using BLASTP against the RefProt database. Best hits were selected by choosing the alignment with the lowest scoring e-value using an e-value threshold of ≤1e^−10^ (if the lowest scoring e-value is the same for multiple species, then multiple hits are shown).
(XLS)

Dataset S4 **Excel spreadsheet of Pfam and Smart domain annotations.** Domains were mapped to *S. mediterranea* sequences using RPS-BLAST; domain hits with e-values≤1e^−3^ were accepted if ≥80% of the domain length mapped to the *S. mediterranea* sequence.
(XLS)

Dataset S5 **Excel spreadsheet of transcript expression profiles in sexual and asexual non-irradiated and irradiated samples; raw expression data are shown as FPKM values and normalized expression values are included as Z-scores.**
(XLS)

Dataset S6 **Excel spreadsheet of transcript expression profiles in X1, X2 and Xins cell populations shown in** **Figure 4A** **; raw expression data are shown as FPKM values.**
(XLS)

Dataset S7 **Excel spreadsheet of** ***S. mediterranea*** **-Human orthologs.**
(XLS)

Dataset S8 **Excel spreadsheet of transcript expression in human embryonic stem cells (hESCs) and day-6 embryoid bodies (hEB); raw expression data are included as FPKM values and expression ratios between cell populations are also shown.**
(XLS)

Dataset S9 **Excel spreadsheet containing transcript expression values for sexual and asexual non-irradiated samples shown in** **Figure 2A** **.** Raw expression data for sexual (Sex_NIR) and asexual (Asex_NIR) animals are shown as FPKM values; strain-specific biases are listed in column 4.
(XLS)

Figure S1 **Effects of irradiation on** ***S. mediterranea*** **adult cell populations.** (A) Fold-change in cell count in non-irradiated (blue) and irradiated (red) X1, X2 and Xins cell populations in asexual and (B) sexual animals.
(PDF)

Figure S2 **RT-PCR Validation.** RT-PCR validation of transcripts in non-irradiated (NIR) and irradiated (IR) sexual and asexual animals is shown for 32 candidates. Band sizes (bps) are indicated for 6 alternatively spliced genes. *Smed-actin* used as a control.
(PDF)

Table S1 Read counts for mRNA-Seq libraries. Libraries for sexual non-irradiated (Sexual NIR) and irradiated (Sexual IR) samples, asexual non-irradiated (Asexual NIR) and irradiated (Asexual IR) samples and *Smed-ago2(RNAi)*, *Smedwi-2(RNAi)* and *Smedwi-3(RNAi)* samples were sequenced as single reads (SR) and/or paired-ends (PE), as specified.
(DOC)

Table S2 Transcript nucleotide composition. The frequency of A, C, G and T nucleotides in the coding sequence of transcripts with FPKM>1; percentages are included in the last column.
(DOC)

Table S3 Most abundant Pfam domain annotations. Top 20 most frequently observed Pfam domains for transcripts with FPKM>1. Domain counts (column 2) and the relative frequency of each domain (column 3) was tabulated using the total number of domain hits. The biological function of each domain is described in the last column.
(DOC)

Table S4 *S. mediterranea*-Human orthologs expressed in X1 neoblast and embryonic stem cell populations.
(XLS)

Table S5 *S. mediterranea*-Human orthologs expressed in irradiation-insensitive Xins and hEB cell populations.
(XLS)

Table S6 Primer sequences used for RT-PCR validation experiments.
(XLS)
