# Supplementary figures and images for: Transcriptome Analysis Reveals Strain-Specific and Conserved Stemness Genes in Schmidtea mediterranea
Source: PLoS One. 2012 Apr 4;7(4):e34447. doi: 10.1371/journal.pone.0034447 (PMC3319590; doi:10.1371/journal.pone.0034447)

Figure S1

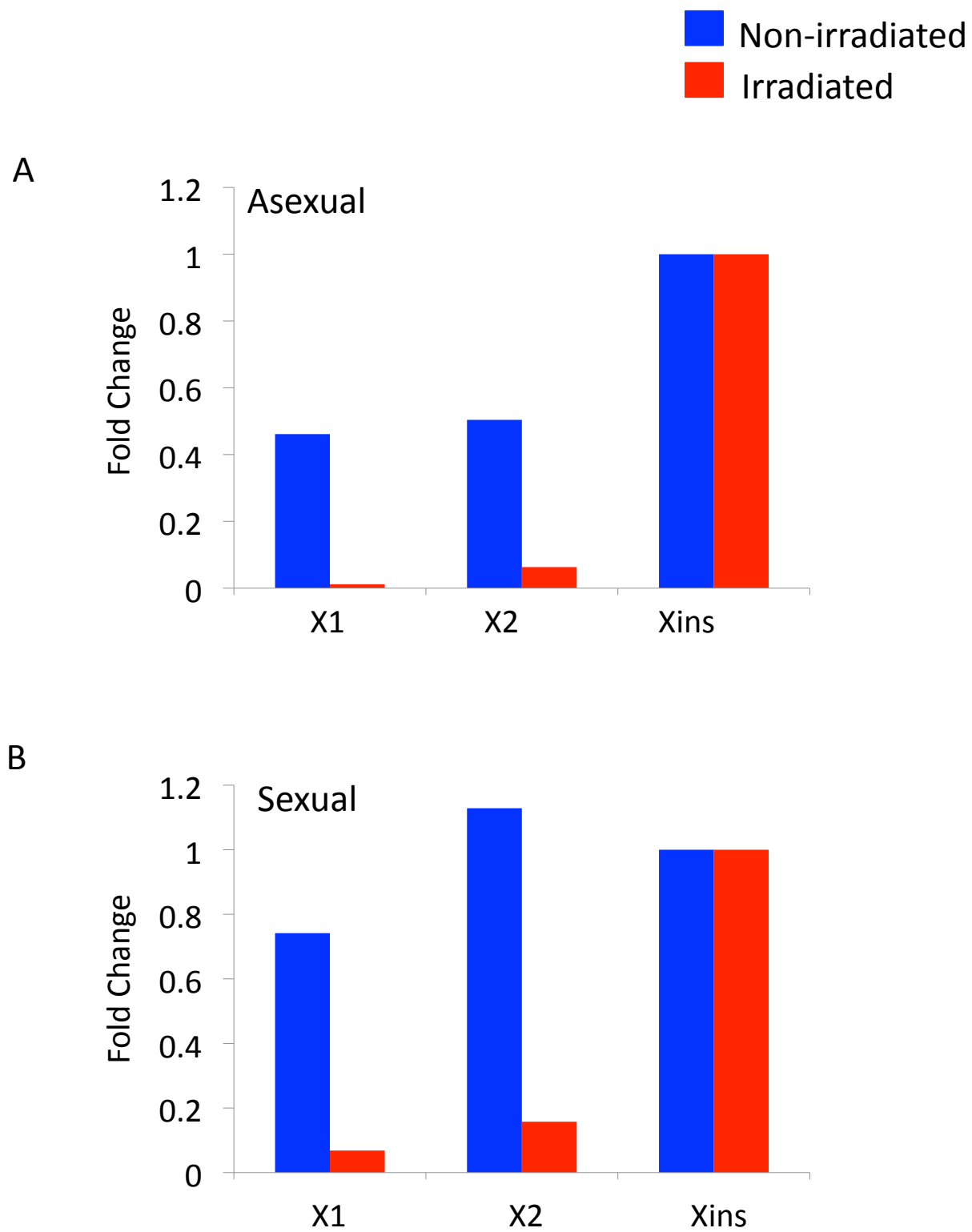

Supplement: Figure S1 — Effects of irradiation on S. mediterranea adult cell populations. (A) Fold-change in cell count in non-irradiated (blue) and irradiated (red) X1, X2 and Xins cell populations in asexual and (B) sexual animals. (PDF) [file pone.0034447.s010.pdf]

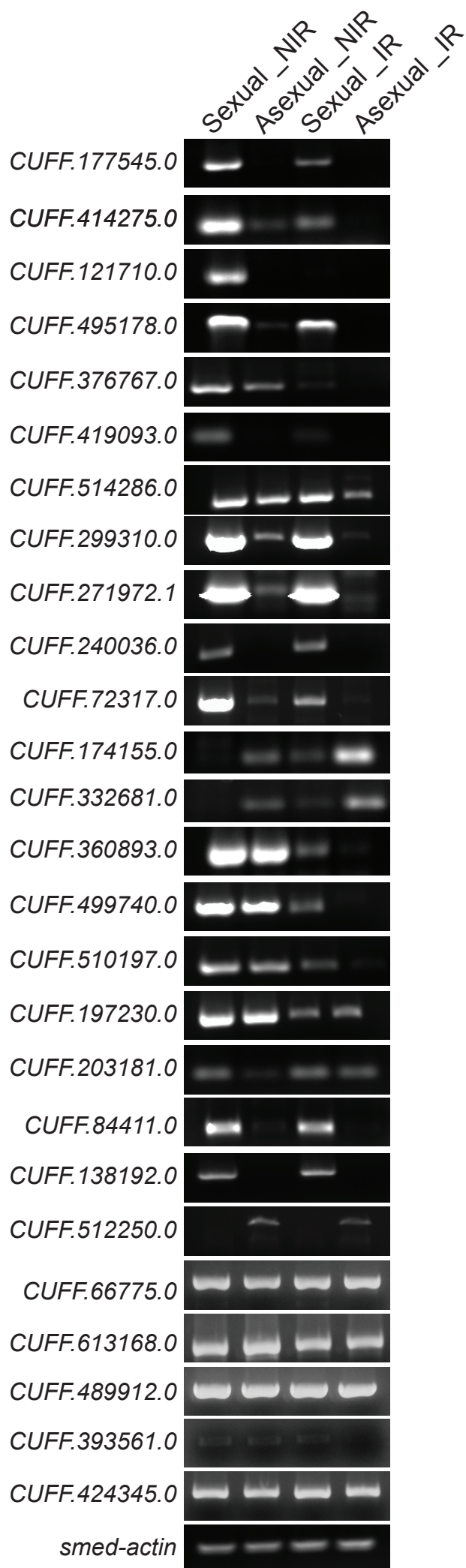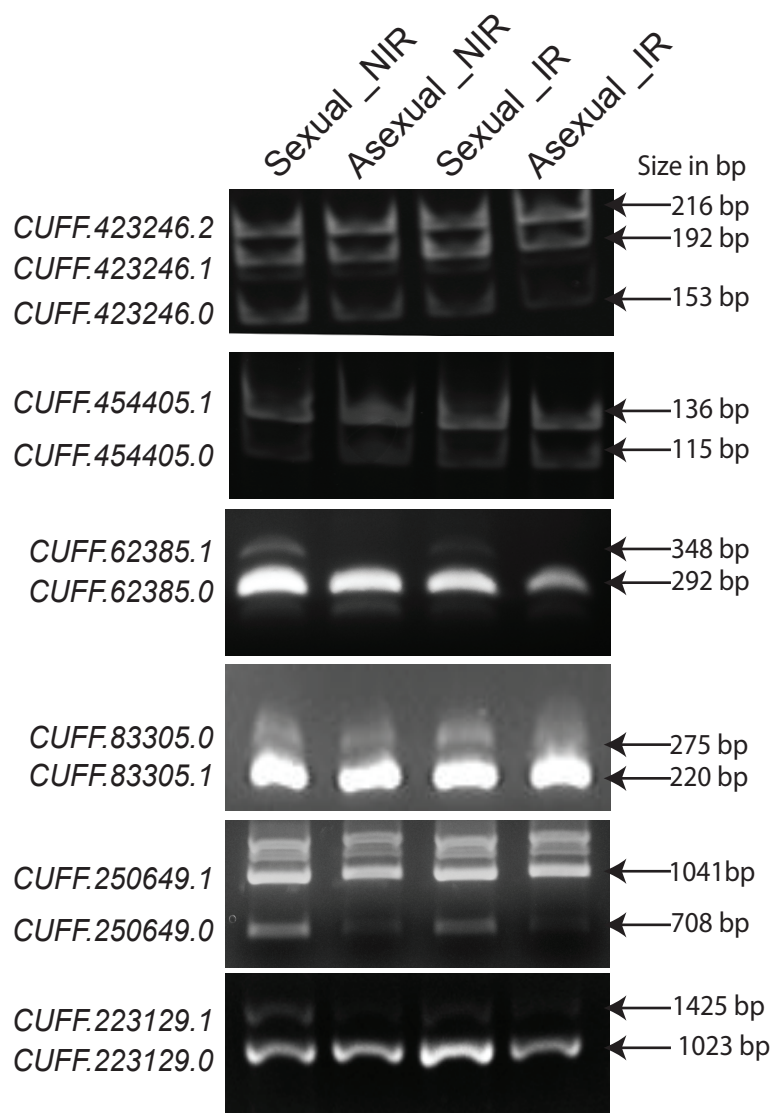

Supplement: Figure S2 — RT-PCR Validation. RT-PCR validation of transcripts in non-irradiated (NIR) and irradiated (IR) sexual and asexual animals is shown for 32 candidates. Band sizes (bps) are indicated for 6 alternatively spliced genes. Smed-actin used as a control. (PDF) [file pone.0034447.s011.pdf]
