# Supplementary material for: Transcriptome Analysis Reveals Strain-Specific and Conserved Stemness Genes in Schmidtea mediterranea
Source: PLoS One. 2012 Apr 4;7(4):e34447. doi: 10.1371/journal.pone.0034447 (PMC3319590; doi:10.1371/journal.pone.0034447)
Supplement: Table S1 — Read counts for mRNA-Seq libraries. Libraries for sexual non-irradiated (Sexual NIR) and irradiated (Sexual IR) samples, asexual non-irradiated (Asexual NIR) and irradiated (Asexual IR) samples and Smed-ago2(RNAi), Smedwi-2(RNAi) and Smedwi-3(RNAi) samples were sequenced as single reads (SR) and/or paired-ends (PE), as specified. (DOC) [file pone.0034447.s012.doc]

Table S1. Read and splice junction counts for RNA-Seq libraries

| **Library** | **Read Length(bp)** | **Total Number Reads** | **Mapped Reads** |
| --- | --- | --- | --- |
| Sexual NIR (SR+PE) | 36,40,37 | 21,524,351 | 13,042,544 |
| Sexual IR (SR) | 36,40 | 13,613,661 | 10,236,994 |
| Asexual NIR (SR+PE) | 36,40,37 | 15,625,957 | 10,911,578 |
| Asexual IR (SR) | 36,40 | 13,346,485 | 10,422,606 |
| *Smedwi-2* RNAi (PE) | 76 | 13,304,568 | 9,495,642 |
| *Smedwi-3* RNAi (PE) | 76 | 12,663,200 | 9,318,755 |
| *Smed-Ago* RNAi (PE) | 37 | 6,078,906 | 4,038,158 |
