# Supplementary material for: Transcriptome Analysis Reveals Strain-Specific and Conserved Stemness Genes in Schmidtea mediterranea
Source: PLoS One. 2012 Apr 4;7(4):e34447. doi: 10.1371/journal.pone.0034447 (PMC3319590; doi:10.1371/journal.pone.0034447)
Supplement: Table S2 — Transcript nucleotide composition. The frequency of A, C, G and T nucleotides in the coding sequence of transcripts with FPKM>1; percentages are included in the last column. (DOC) [file pone.0034447.s013.doc]

Table S2. Transcript nucleotide composition

|  | **Total (nts)** | **Percentage** |
| --- | --- | --- |
| A | 6,128,918 | 34% |
| C | 3,102,977 | 17% |
| G | 3,425,089 | 19% |
| T | 5,504,498 | 30% |
