# Supplementary material for: Transcriptome Analysis Reveals Strain-Specific and Conserved Stemness Genes in Schmidtea mediterranea
Source: PLoS One. 2012 Apr 4;7(4):e34447. doi: 10.1371/journal.pone.0034447 (PMC3319590; doi:10.1371/journal.pone.0034447)
Supplement: Table S3 — Most abundant Pfam domain annotations. Top 20 most frequently observed Pfam domains for transcripts with FPKM>1. Domain counts (column 2) and the relative frequency of each domain (column 3) was tabulated using the total number of domain hits. The biological function of each domain is described in the last column. (DOC) [file pone.0034447.s014.doc]

Table S3. Most abundant Pfam domain annotations

| **Pfam Domain** | **Domain Count** | **Relative Frequency** | **Biological Function** |
| --- | --- | --- | --- |
| Ankyrin | 185 | 0.045 | Transcription, cell-cycle regulation, ion transport, signal transduction |
| Pkinase | 83 | 0.020 | Protein amino acid phosphorylation |
| RRM | 77 | 0.019 | RNA-binding |
| WD40 | 67 | 0.016 | Signal transduction, transcription, cell-cycle control, apoptosis |
| SH3 | 62 | 0.015 | Protein-protein interactions, signal transduction |
| Pkinase_Tyr | 62 | 0.015 | Cellular division, proliferation, differentiation, apoptosis |
| I-set | 61 | 0.015 | Cell-cell recognition, muscle structure and immune response |
| Helicase_C | 60 | 0.015 | Couples ATPase activity to RNA binding and unwinding |
| Cadherin | 52 | 0.013 | Ca2+ dependent homophilic cell adhesion, regulation of morphogenesis and regeneration |
| PDZ | 52 | 0.013 | Signaling transduction |
| C2 | 45 | 0.011 | Ca2+ dependent membrane trafficking, signal transduction |
| SH3_2 | 43 | 0.011 | Cytoskeletal organization, signal transduction |
| SH2 | 40 | 0.010 | Regulates intracellular signaling cascades |
| Homeobox | 37 | 0.009 | DNA-binding |
| Tubulin_C | 37 | 0.009 | GTPases involved in polymer formation |
| Miro | 34 | 0.008 | Rho GTPases involved in mitochondrial homeostasis and apoptosis |
| Ras | 32 | 0.008 | Ras GTPases that regulate cell growth, survival, differentiation, chemotaxis, vesicle-trafficking |
| DEAD | 29 | 0.007 | Unwinding nucleic acids, RNA metabolism, pre mRNA splicing, transcription, translation, RNA decay |
| PHD | 29 | 0.007 | DNA/RNA/protein-binding, chromatin remodeling, transcription, translation, mRNA trafficking |
| Fn3 | 28 | 0.007 | Cell adhesion, cell differentiation and migration, cytoskeletal maintenance |
